# Enhancing the therapeutic potential of FDA-approved ifosfamide and 5-fluorouracil through rational chemical modifications for endometrial cancer treatment

**DOI:** 10.1007/s12032-025-02821-2

**Published:** 2025-06-25

**Authors:** Laiba Tanveer, Salma Batool, Hira Mubeen, Izzah Shahid

**Affiliations:** 1 Department of Biotechnology, Faculty of Science & Technology, University of Central Punjab, Lahore, Pakistan; 2 Department of Basic and Applied Chemistry, Faculty of Science & Technology, University of Central Punjab, Lahore, Pakistan; 3 Institute of Multidisciplinary Research in Applied Biology, Public University of Navarra, Pamplona, Spain

**Keywords:** Ifosfamide, KRAS, Ligand functionalization, Uterine cancer, 5-Fluorouracil

## Abstract

Endometrial or uterine cancers predominantly occur in postmenopausal women, thereby establishing a strong correlation with advanced age. The chemotherapeutic agents ifosfamide and 5-fluorouracil have demonstrated notable efficacy against endometrial cancer by inhibiting cell division and proliferation. Nevertheless, current pharmacological treatments encounter challenges related to drug resistance and adverse effects. To assess the potential of drug modifications, in silico methodologies were employed to alter the molecular structures of ifosfamide and 5-fluorouracil with the aim of enhancing their efficacy and binding affinity to type 1 endometrial cancer. The three-dimensional configurations of mutated KRAS proteins were sourced from the Protein Data Bank. The molecular structures of ifosfamide and 5-fluorouracil were obtained from PubChem and EMBL-EBI, and subsequently modified using Chemsketch. The modifications included the addition of a methyl group, benzene ring, nitrogen atom, cyclopentane ring, and fluorine atoms to the drugs. The binding affinities of these modified drugs to the proteins were visualized using AutoDock Vina. The modified drugs exhibited improved binding affinities of −7.4 and −7.5 kcal/mol with both mutated target proteins. SwissADME analysis and Molinspiration’s evaluation of Lipinski’s rule parameters suggested that these modified drugs hold promise for the treatment of type 1 endometrial cancer, pending preclinical and clinical trials. This study represents a significant advancement in drug modification as a potential chemotherapeutic strategy for endometrial cancer. Among the two modified drugs, altered 5-fluorouracil demonstrated superior binding affinity and pharmacokinetic properties, rendering it a promising candidate.

## Introduction

Uterine cancer is one of the common and frequently diagnosed type of gynecological cancers [1]. Uterine cancer emerging from endometrium is referred to as endometrial cancer which is ranked as the sixth most common type of cancer among females worldwide [2]. In 2020 alone, approximately 97,000 women lost their lives to this cancer, accounting for 3% of all female cancer deaths [3]. Usually, endometrial cancer is considered to be the cancer of old age as it is mostly diagnosed in post-menopausal women accounting for nearly 75 to 85% of the cases, while 20 to 25% of patients develop uterine cancer before menopause [4–6]. The median age at which endometrial cancer is diagnosed is 63 years [7].

Endometrial cancer has two subtypes: type 1 and type 2 [8]. Type 1, also known as endometrioid endometrial cancer or endometrioid adenocarcinomas is a low-grade tumor which occurs as a result of unopposed exposure to estrogen [9]. Endometrioid endometrial cancer is a common type of uterine cancer accounting for approximately 65 to 80% cases [10]. Type 2 cancer or non-endometrioid endometrial cancer is a non-estrogen-dependent cancer and is referred as serous, clear, and squamous cell carcinomas [11]. Type 2 cancer is a rare and aggressive type accounting for approximately 10 to 20% of uterine cancers [12]. Type 1 cancer occurs as a result of mutation in PTEN, β-catenin, and *KRAS* gene and also due to microsatellite instability while Type 2 occurs due to mutation in *TP53* and HER2/neu genes [13, 14]. The TP53 protein maintains genomic stability by regulating cell cycle arrest, DNA repair, apoptosis, and senescence in response to cellular stress. In endometrial cancer, p53 alterations are linked to aggressive, high-grade tumors, particularly serous carcinoma with poor prognosis [15]. Key mutations in the DNA-binding domain include R175H causing protein misfolding, R248Q affecting DNA-binding, and R273H impairing transcriptional functions [16]. In endometrial cancer, gain-of-function mutations promote tumor progression through EGFR/PI3K/AKT signaling and EMT, leading to metastasis, treatment resistance, and chromosomal instability that triggers cGAS-STING pathway and immune evasion [17].

Kirsten Rat Sarcoma Viral Oncogene Homolog (KRAS) mutations are the most prevalent in Type 1 endometrial cancer. KRAS, a member of the RAS family of small GTPases, acts as a molecular switch in cell proliferation and survival signaling pathways. Mutations in KRAS, especially in codon 12, result in the constant activation of downstream pathways like MAPK and PI3K/AKT, leading to uncontrolled cell growth and tumorigenesis in endometrial cancer. Understanding the molecular basis of KRAS mutations in endometrial cancer opens up opportunities for targeted therapeutic interventions and the development of novel chemotherapeutics with enhanced efficacy [18].

Chemotherapy is considered the best option for treating uterine cancer. Chemotherapeutics includes platinum-based drugs, anthracycline, pyrimidines, taxanes, inhibitors, antineoplastic, and alkylating agents used to treat uterine cancer [19, 20]. These drugs inhibit tumor cell growth; however, prolonged use of chemotherapeutic agents has led to resistance development due to genetic mutations, drug efflux, DNA repair mechanisms, epigenetic changes, target alteration, and signaling pathway activation [21, 22]. Chemotherapeutic drugs have cytotoxic side effects on patients, killing normal cells and suppressing the immune system [23]. As endometrial cancer causes significant mortality, there is a need to discover new drugs or develop improved ones with better response rates and efficacy. This in silico study was designed to tackle the challenge of emerging drug resistance in endometrial cancer treatment by modifying existing drugs through drug prediction methodologies to develop a novel therapeutic option. Bioinformatics tools were employed to modify the structures of ifosfamide and 5-fluorouracil, aiming to enhance their efficacy and reduce the resistance developed against them. The successfully modified drugs were then compared to identify the most suitable candidate for treating Type 1 endometrial cancer. The pharmacological and protein-binding properties of the modified drug compounds were evaluated using in silico analysis, focusing on their ability to penetrate the gastrointestinal tract. The modifications aimed to enhance drug efficacy while preserving optimal pharmacokinetics, paving the way for future research.

## Material and methods

### Identification and preparation of protein

Three-dimensional structures of guanosine diphosphate (GDP)-bound G12C oncogenic mutant of human GTPase KRAS protein (PDB ID; 4LDJ) and G12C oncogenic variant of human KRAS protein (PDB ID; 4NMM) were retrieved in PDB format from Protein Data Bank (https://www.rcsb.org/). Protein Data Bank (PDB) is a significant database which contains three-dimensional (3D) atomic structures of biomolecules, including proteins and nucleic acids. Protein Data Bank (PDB) provides a detailed description of both the normal and mutated protein structures. These structures were selected due to their relevance in Type 1 endometrial cancer, where KRAS mutations are commonly implicated. Using BIOVIA Discovery Studio Visualizer and AutoDock tools (https://discover.3ds.com/), standard preparation steps were followed, such as co-crystallized ligands and water molecules were removed to avoid non-specific interactions, polar hydrogens were added, and Kollman charges were assigned to ensure accurate modeling of electrostatic interactions. The protein structures were then energy minimized using Discovery Studio’s default protocol to relieve steric clashes and optimize geometry. To prepare the files for docking with AutoDock Vina, the structures were converted to PDBQT format, which encodes torsion flexibility and partial charges. The binding site was defined based on literature and focused around residue 12, which is the site of the G12C mutation, using grid box coordinates specific to each protein. This preparation ensured the proteins were in a suitable conformation and chemical state for reliable molecular docking analysis [24].

### Identification and preparation of ligand

Different leading chemotherapeutic drugs used for treating uterine cancer were identified with the help of literature review. Three-dimensional (3D) structures of cordycepin (C_10_H_13_N_5_O_3_), cyclophosphamide (C_7_H_15_Cl_2_N_2_O_2_P), 5-fluorouracil (C_4_H_3_FN_2_O_2_), ifosfamide (C_7_H_15_Cl_2_N_2_O_2_P), carboplatin (C_6_H_12_N_2_O_4_Pt), paclitaxel (C_47_H_51_NO_14_), doxorubicin (C_27_H_29_NO_11_), topotecan (C_23_H_23_N_3_O_5_), temsirolimus (C_56_H_87_NO_16_), and gemcitabine (C_9_H_11_F_2_N_3_O_4_) were retrieved in Structure Data Format (SDF) from PubChem available at (https://pubchem.ncbi.nlm.nih.gov/). PubChem is a database containing information about the chemical structures, properties, and biological activities of ligands or chemical molecules. Later on, BIOVIA Discovery Studio Visualizer was used to convert three-dimensional (3D) structures into Protein Data Bank (PDB) format [25]. Two-dimensional (2D) structures of 5-fluorouracil and ifosfamide were retrieved from EMBL-EBI in Molfile format. EMBL-EBI (https://www.ebi.ac.uk/chebi/) database provides up-to-date information about drug-like small molecules and their targets.

### Screening of ligands

The selected library of chemotherapeutic agents was initially screened by docking against the mutant KRAS proteins (PDB IDs: 4LDJ and 4NMM). Molecular docking was performed using AutoDock Vina (https://vina.scripps.edu/), a widely used docking software that predicts the preferred orientation of ligands when bound to a protein receptor, estimating binding affinities through an efficient scoring function. Ligands were energy minimized and converted to the appropriate format (PDBQT) compatible with AutoDock Vina. The docking process involved flexible ligand docking into a rigid receptor to evaluate potential interactions and binding energies. Ligands demonstrating the lowest binding energy (indicative of higher binding affinity) were shortlisted for further structural modification. The screening was performed to identify promising candidates for rational drug design aimed at overcoming drug resistance in Type 1 endometrial cancer. The binding sites were identified based on prior structural knowledge and literature reports focusing on the mutation hotspots in KRAS, particularly residue 12, which is critical for its oncogenic activity. A grid box was centered around this residue with dimensions sufficient to encompass the active site and nearby residues potentially involved in ligand binding. This ensured that docking simulations focused on the biologically relevant binding pocket to provide meaningful interaction data [26].

### Ligand functionalization

Chemsketch (https://www.acdlabs.com/) is an offline tool allowing to draw chemical structures, reactions, and diagrams. The selected structures with low-binding affinities were modified using Chemsketch. Methyl group and benzene ring were added at carbon-4 of 5-fluorouracil [27, 28]. At carbon-3 of the additional benzene ring, nitrogen atom and a methyl group were added, whereas at carbon-4 and carbon-5, fluorine atoms were added. Nitrogen atom at carbon-3 of ifosfamide was replaced by a methyl group and chlorine atom was replaced with a cyclopentane ring.

### Protein–ligand interaction analysis

Normal and modified ifosfamide and 5-fluorouracil were docked with 4NMM and 4LDJ using Autodock Vina. Site-specific docking was carried out, and the grid box was set at residue number 12 of the mutated proteins [29]. For 4LDJ, the center coordinates x, y, and z were set as 48.163, 27.929, and 12.71, respectively. While for 4NMM, the center coordinates x, y, and z were set as 47.909, 30.533, and 10.198, respectively. Interactions between the ligands and the proteins were visualized using PyMOL (https://pymol.org/). The bond length was observed to identify the bonds formed between the protein and the modified drug.

### Simulation analysis of complex

Molecular Dynamics (MD) simulations were conducted using the iMODS server (http://imods.iqfr.csic.es/), an online platform that performs internal coordinate normal mode analysis (NMA) for protein–ligand complexes to evaluate their conformational stability and flexibility. The protein–ligand complexes, obtained from docking (mutant KRAS proteins with modified Ifosfamide and 5-Fluorouracil), were uploaded in PDB format, and simulations were executed using the elastic network model (ENM)-based normal mode analysis (NMA). The AMBER94 force field parameters were automatically applied to approximate interatomic interactions within the system. Each complex consisted of approximately 3500 to 4200 atoms, depending on the ligand and protein structure. Simulations were performed in a vacuum environment (dry system, without explicit water molecules), consistent with the modeling scope of iMODS, which focuses on estimating intrinsic mobility rather than solvent-mediated dynamics. Normal mode analysis (NMA) was used to estimate deformability, eigenvalues, B-factors, covariance maps, and the elastic network model, collectively representing the system’s dynamic behavior. Although iMODS does not explicitly simulate time evolution in femtoseconds or nanoseconds like traditional MD, the eigenvalues and mobility vectors provide indirect insights into the energy required to deform the complex and the flexibility of various regions. The deformability graphs and covariance matrices generated indicate the relative flexibility of the protein–ligand complex, where lower eigenvalues suggest greater stability. These simulation results were used to validate the docking outcomes and assess the dynamic behavior of the modified drugs in the active site of KRAS mutant proteins [30, 31].

### In silico pharmacokinetic and drug-likeness evaluation

Pre-clinical testing and ADMET analysis of modified ligands was done through SwissADME (http://www.swissadme.ch/). SwissADME is an online tool used to evaluate and predict the absorption, distribution, metabolism, and excretion (ADMET) properties, physicochemical properties, pharmacokinetics, and permeability of small drug molecules. The two-dimensional (2D) structure of the compound was included in the input and its drug-likeness qualities were determined [32].

### Validation of Lipinski’s rule of five

The drug-like qualities of the improved molecule were investigated using the Molinspiration cheminformatics (https://www.molinspiration.com/cgi/properties). It helps in manipulation and processing of molecules, calculation of different molecular properties, modeling and drug design, three-dimensional (3D) structure generation, and bioactivity prediction. The modified structure of the compound was drawn as input and the molecular properties were determined.

## Results

### Screening of ligands

Molecular docking is used to determine interactions between a ligand and the target protein. The resultant binding affinity determines the potential of a small ligand to bind to the protein, with a lower binding energy indicating a stronger interaction and making it a favorable drug candidate. Autodock Vina was used for screening all the retrieved ligands. Table 1 shows the binding affinities of the selected ligand molecules. Among selected ligands, the highest binding affinity was shown by cordycepin with both proteins followed by gemcitabine. The lowest binding affinity was indicated by Ifosfamide in both of the cases. Binding affinity of 5-fluorouracil was also insignificant as compared to other molecules. Based on the extensive use, 5-fluorouracil and ifosfamide with the comparatively fewer binding affinities were selected for further structural modification.

**Table 1:** Binding affinity of the selected ligands with 4NMM and 4LDJ proteins

Proteins (PDB codes)	Ligands	Binding affinities (kcal/mol)
4NMM	Cordycepin	−6.6
	Cyclophosphamide	−4.7
	**5-Fluorouracil**	−**5.3**
	**Ifosfamide**	−**4.6**
	Carboplatin	−5.8
	Gemcitabine	−5.9
4LDJ	Cordycepin	−6.6
	Cyclophosphamide	−4.9
	**5-Fluorouracil**	−**5.4**
	**Ifosfamide**	−**5.0**
	Carboplatin	−5.8
	Gemcitabine	−6.4

Bold indicates Lower binding affinities displayed by 5-fluorouracil and ifosfamide with target proteins

### Structure modification and interaction studies

**Fig. 1:**
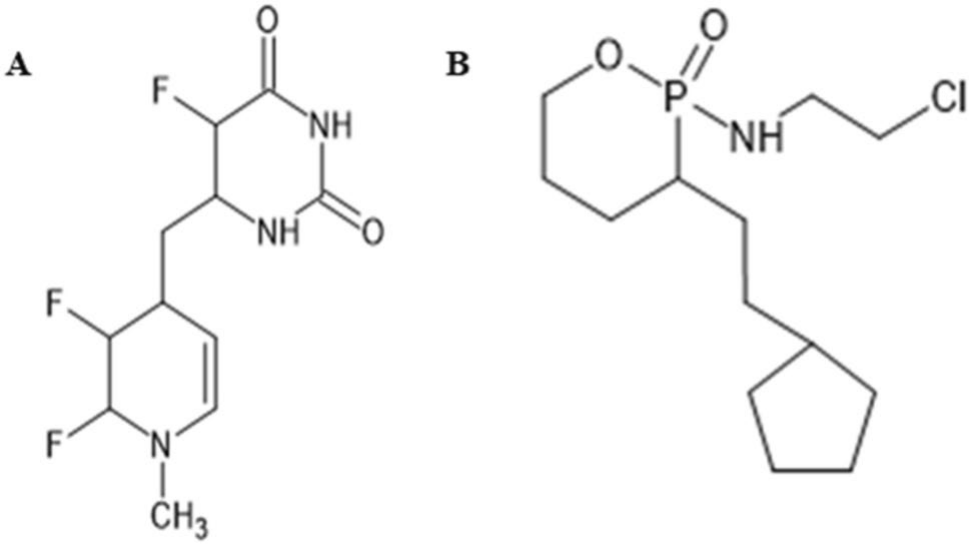
Addition of methyl and benzene groups at carbon-4, addition of nitrogen atom, methyl group at carbon-3, and fluorine atoms at carbon-4 and carbon-5 of additional benzene ring in 5-fluorouracil (**A**) and replacement of nitrogen atom at carbon-3 with a methyl group and chlorine atom with cyclopentane ring (**B**)

Among selected ligands for structural modification, 5-fluorouracil was successfully modified by the addition of a methyl group and a benzene ring at C-4 as indicated in Fig. 1A. Another modification was done at C-3 of the additional benzene ring, where a nitrogen atom and at C-4 and C-5, fluorine atoms were added. In case of ifosfamide, modification was made by replacing nitrogen atom at C-3 of ifosfamide with a methyl group and chlorine atom with a cyclopentane ring (Fig. 1B). Binding affinity of modified 5-fluorouracil improved from −5.4 to −7.5 kcal/mol with 4LDJ protein. While, the energy absorption of modified 5-fluorouracil with 4NMM was improved from −5.3 to −7.4 kcal/mol. Energy absorption of ifosfamide was increased from −4.6 to −6.5 kcal/mol with 4NMM protein. While, the binding affinity of ifosfamide was improved from −5.0 to −6.6 kcal/mol with 4LDJ protein as shown in Table 2.

**Table 2:** Binding affinity of normal and modified 5-fluorouracil and ifosfamide with 4NMM and 4LDJ

Ligands	Ligand type	Binding affinities (kcal/mol) with 4NMM	Binding affinities (kcal/mol) with 4LDJ
5-fluorouracil	Normal	−5.3	−5.4
5-fluorouracil + methyl group + benzene ring + nitrogen atom + fluorine atoms	Modified	−7.4	−7.5
Ifosfamide	Normal	−4.6	−5.0
Ifosfamide + methyl group + cyclopentane	Modified	−6.5	−6.6

### Protein–ligand interaction analysis

**Fig. 2:**
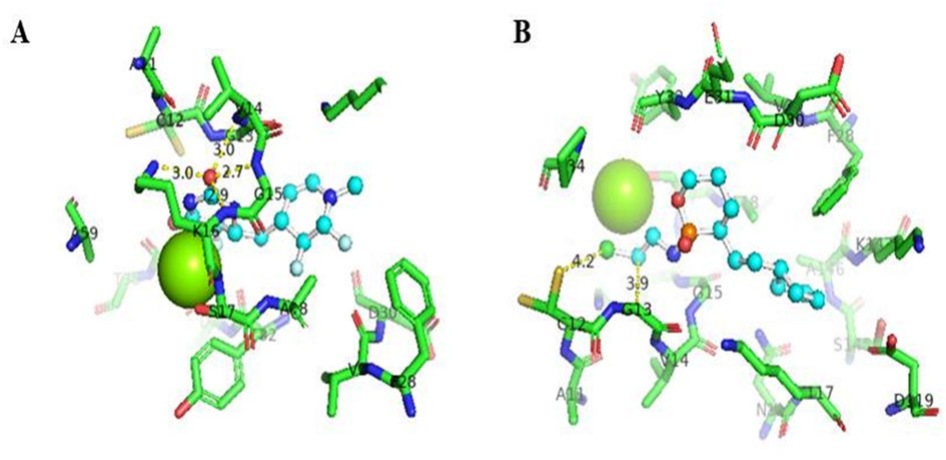
Interaction between modified 5-fluorouracil and 4NMM protein (**A**) and interaction between modified 5-fluorouracil and 4LDJ protein (**B**)

**Fig. 3:**
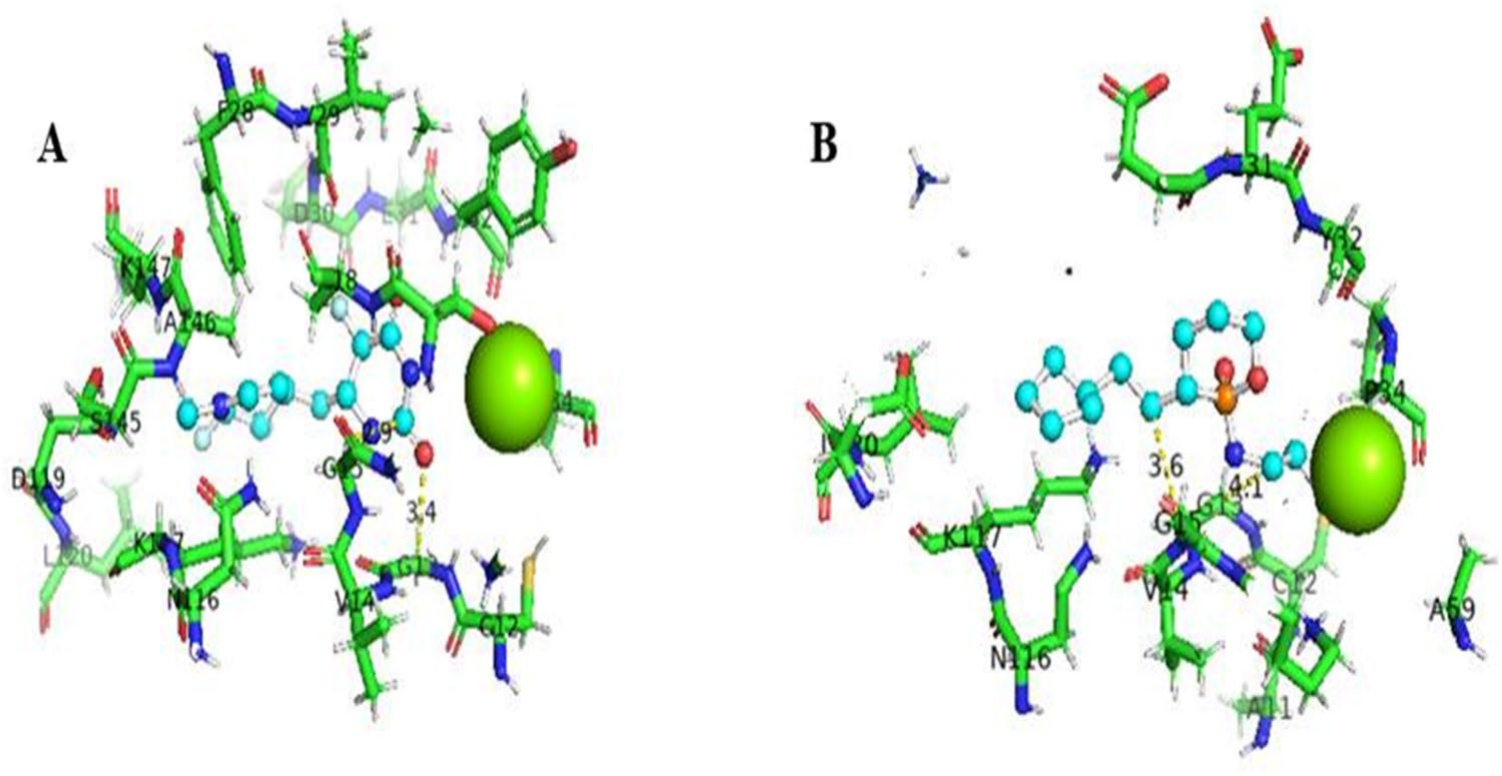
Interaction between modified ifosfamide and 4NMM protein (**A**) and interaction between modified ifosfamide and 4LDJ protein (**B**)

The interactions between the protein and the ligand were interpreted by PyMOL to visualize the conventional bonds with the help of the bond length. Four hydrogen bonds with the bond lengths of 3.0, 3.0, 2.9, and 2.7 Å were observed in the complex of 4NMM with 5-fluorouracil (Fig. 2A). Only one Van der Waals or hydrophobic bond with the bond length of 3.6 Å was predicted in 4LDJ and 5-fluorouracil complex (Fig. 2B). The bond length of hydrogen bond is in the range of 2.7 to 3.2 Å. Figure 3A shows that one Van der Waals bond with the bond length of 3.9 Å was predicted in the conjugate of 4NMM and ifosfamide as the bond length of Van der Waals bond is in the range of 3.3 to 4.0 Å. In 4LDJ complex with ifosfamide, one Van der Waals bond with the bond length of 3.6 Å was formed (Fig. 3B).

### Simulation analysis of complex

**Fig. 4:**
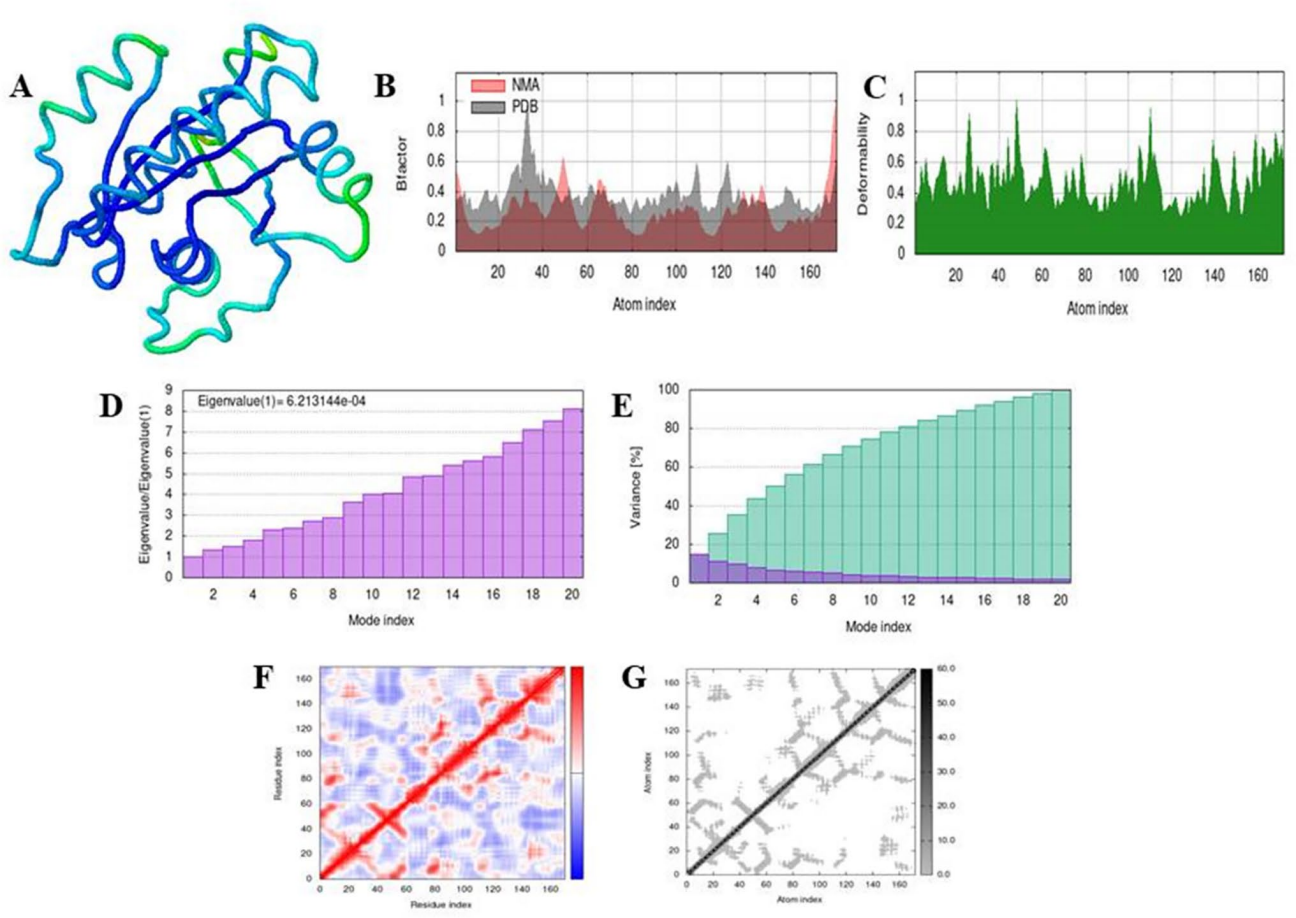
The results of molecular dynamic simulation of 4NMM complex with 5-fluorouracil using iMODS, stimulated 3D structure of the complex with NMA mobility (**A**), B-factor (**B**), deformability (**C**), Eigenvalues (**D**), variance (purple color (individual variance) and green color (cumulative variance)) (**E**), covariance map (red color (correlated motion) and blue color (anti-correlated motion)) (**F**), and elastic network; darker gray region represents stiffer region (**G**)

**Fig. 5:**
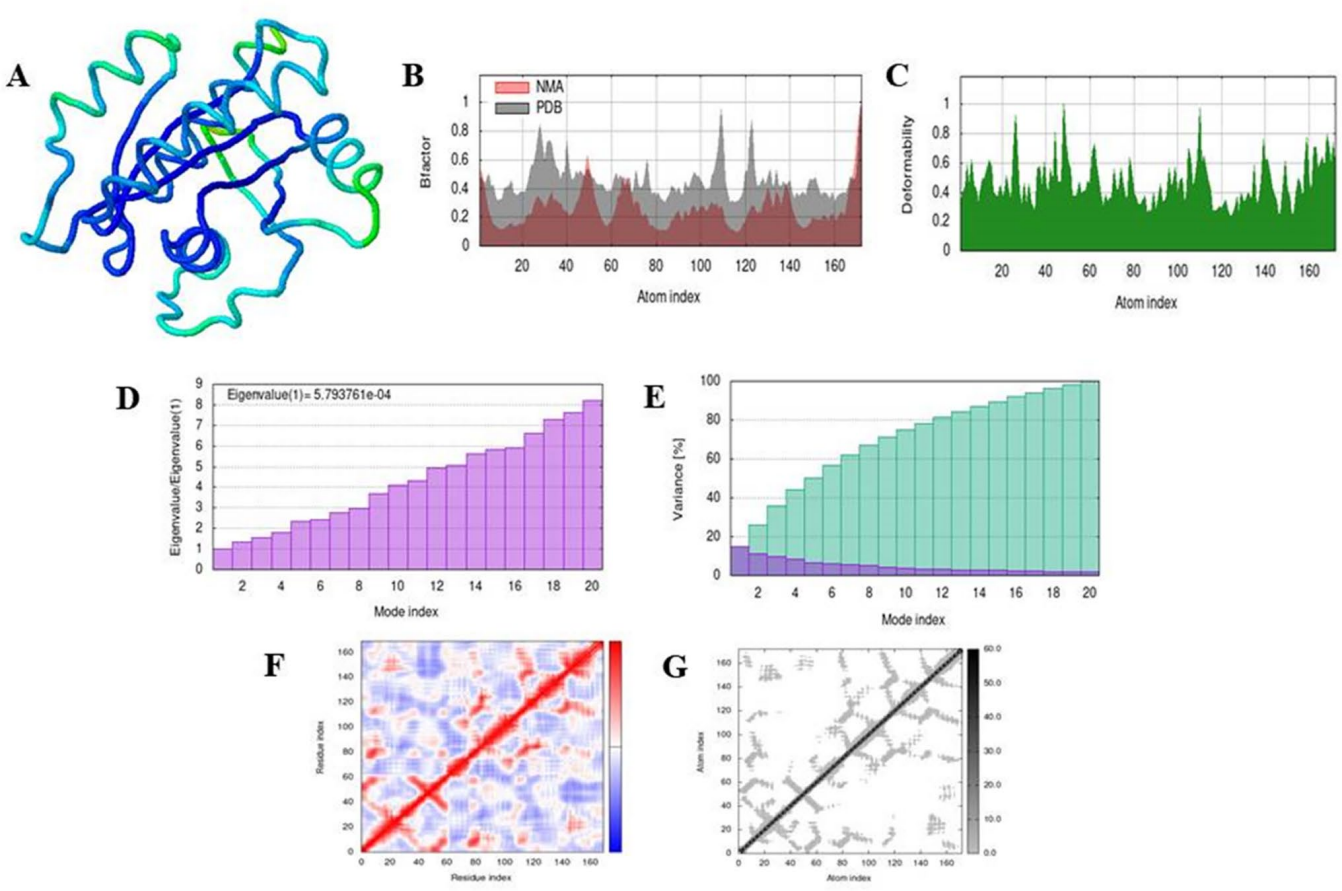
The results of molecular dynamic simulation of docked complex of 4LDJ with 5-fluorouracil using iMODS, stimulated 3D structure of the complex with NMA mobility (**A**), B-factor (**B**), deformability (**C**), Eigenvalues (**D**), variance (purple color (individual variance) and green color (cumulative variance) (**E**), covariance map (red color (correlated motion) and blue color (anti-correlated motion)) (**F**), and elastic network; darker gray region represents stiffer region (**G**)

**Fig. 6:**
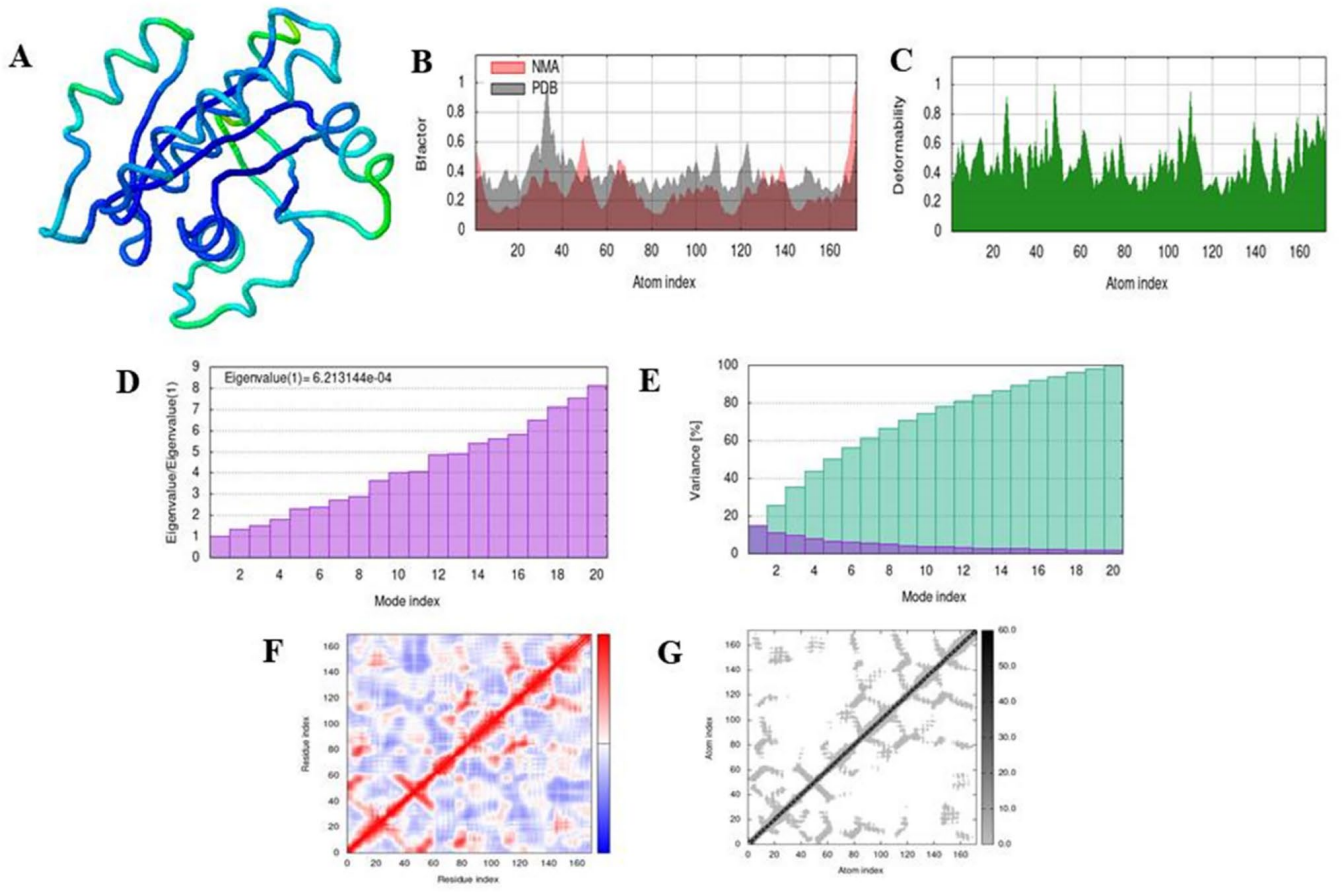
The results of molecular dynamic simulation of docked complex of 4NMM with ifosfamide using iMODS, stimulated 3D structure of the complex with NMA mobility (**A**), B-factor (**B**), deformability (**C**), Eigenvalues (**D**), variance (purple color (individual variance) and green color (cumulative variance) (**E**), covariance map (red color (correlated motion) and blue color (anti-correlated motion)) (**F**), and elastic network; darker gray region represents stiffer region (**G**)

**Fig. 7:**
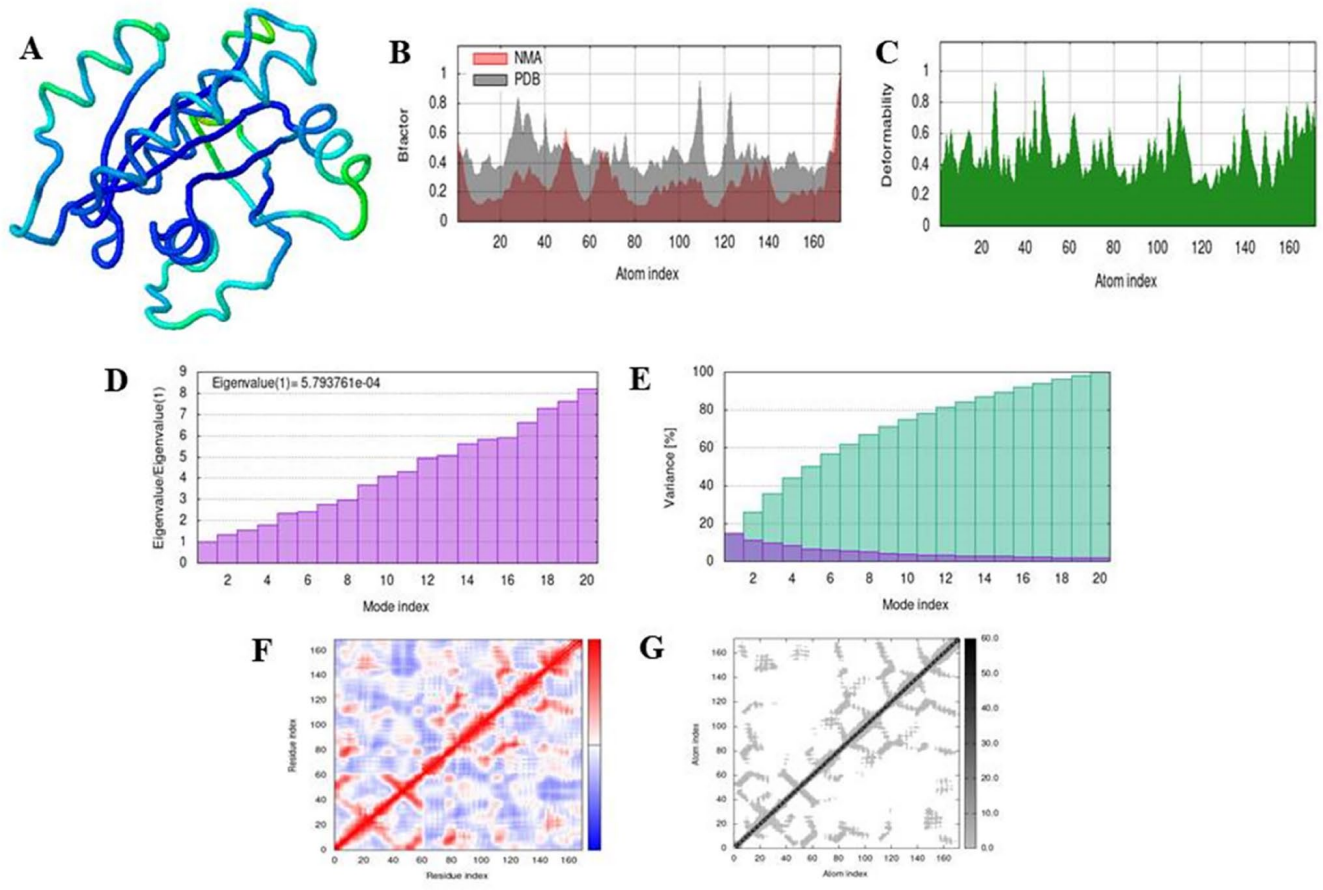
The results of molecular dynamic simulation of docked complex of 4LDJ with ifosfamide using iMODS, stimulated 3D structure of the complex with NMA mobility (**A**), B-factor (**B**), deformability (**C**), Eigenvalues (**D**), variance (purple color (individual variance) and green color (cumulative variance) (**E**), covariance map (red color (correlated motion) and blue color (anti-correlated motion)) (**F**), and elastic network; darker gray region represents stiffer region (**G**)

Different parameters were computed by iMODS for simulation analysis of docked complexes. Figures 4 and 5 show the results of molecular dynamic simulation of docked complexes of 4NMM and 4LDJ with 5-fluorouracil, respectively. Similarly, Figs. 6 and 7 predict the results of docked complexes of 4NMM and 4LDJ proteins with ifosfamide. The two colored affine arrows represented the mobility of domains. The deformability graph showed that minor changes can occur at residues level. The anticipated Eigen value of the complex structure of 5-fluorouracil with 4NMM and ifosfamide with 4NMM was 6.213144e−04 (Figs. 4D and 6D) and that of docked complex of 5-fluorouracil with 4LDJ and ifosfamide with 4LDJ was 5.793761e−04 (Figs. 5D and 7D) which means that high amount of energy will be required to deform both the complexes. Both the Eigen value and the variance maps were inversely related to each other. Co-variance map (Figs. 4F, 5F, 6F, and 7F) showed anti-correlated motion of most of the amino acids of the docked complex of protein with the modified drug which is indicated by blue color on the heat map. Figures 4G, 5G, 6G, and 7G represents the elastic network, with stiffer regions shown by darker gray patches.

### Pre-clinical testing of modified ligands

The modified 5-fluorouracil and ifosfamide were subjected to pre-clinical testing to assess their solubility and interaction with the body tissues to ensure their safety and efficacy. Physicochemical properties, water solubility, gastrointestinal (GI) absorption, skin permeability, blood–brain barrier (BBB) permeation, synthetic accessibility, bioavailability, and topological surface area of drugs were predicted through ADMET analysis. Based on the results of ADMET analysis, the modified 5-fluorouracil and ifosfamide were unable to penetrate the skin. ADMET analysis further confirmed that the modified drugs were not an inhibitor of cytochrome and had optimum lipophilicity. Both the modified 5-fluorouracil and ifosfamide are soluble in water and have high synthetic accessibility, indicating good drug development potential. Moreover, the bioavailability was moderate for both drugs in terms of scoring. Table 3 shows the ADMET parameters of the modified ligands.

**Table 3:** ADMET parameters of pre-clinical testing of modified ligands 5-fluorouracil and ifosfamide

ADMET parameters	Parametric values (5-fluorouracil)	Parametric values (ifosfamide)
Formula Molecular weight Num. heavy atoms Num. arom. heavy atoms Fraction Csp3 Num. rotatable bonds Num. H-bond acceptors Num. H-bond donors Molar Refractivity TPSA (Toplogical Polar Surface Area) Water Solubility Log S (ESOL) GI Absorption BBB Permeation Skin Permeation (Log Kp) Bioavailability Score Synthetic Accessibility Drug-likeness Medicinal Chemistry (Brenk)	C11H14F3N3O2 277.24 g/mol 19 0 0.64 2 5 2 71.48 61.44 Å^2^ −2.18 High NO −7.14 cm/s 0.55 4.58 Yes; 0 violation 1 alert, i.e., N–C-halo	C13H25ClNO2P 293.77 g/mol 18 0 1.00 6 3 1 77.72 48.14 Å^2^ −3.31 High Yes −5.78 cm/s 0.55 5.15 Yes; 0 violation 2 alert, i.e., alkyl_halide, phosphor

### Boiled egg model

**Fig. 8:**
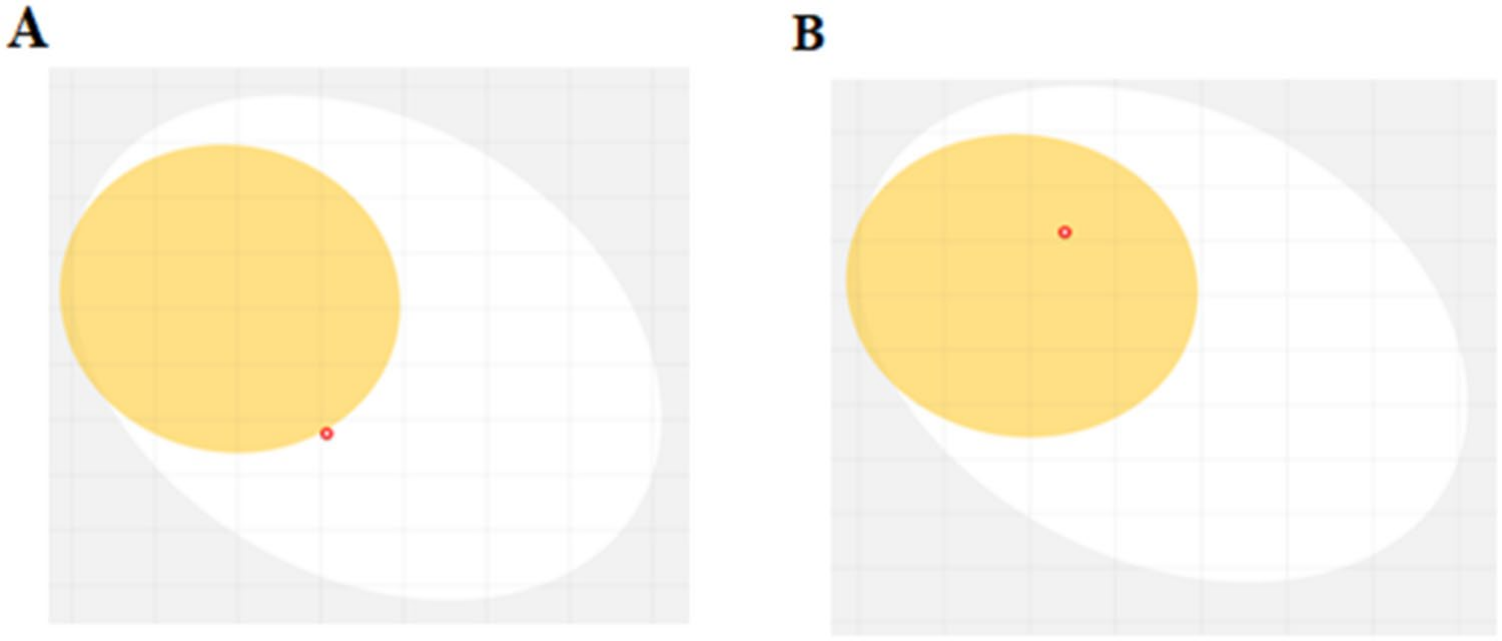
The boiled egg models of modified 5-fluorouracil (**A**) and the boiled egg models of modified ifosfamide (**B**), respectively

The SwissADME server predicted the boiled egg model. The boiled egg model is a reliable method for analyzing how small molecules are absorbed in the gastrointestinal tract and their ability to cross the BBB. This model assigns white or yellow area to indicate gastrointestinal absorption and BBB, respectively. By applying this model, it was found that modified 5-fluorouracil can be effectively absorbed through gastrointestinal tract, while the modified ifosfamide can penetrate the BBB (Fig. 8).

### Validation of Lipinski’s rule of five

Table 4 shows the results of Molinspiration’s estimation of the parameters of Lipinski’s rule for 5-fluorouracil and ifosfamide, including logP (octanol/water partition coefficient), molecular weight, number of hydrogen bond donor (nOHNH)/ acceptor (noN), and the number of rotatable bonds (nrotb**)**. There was no violation of Lipinski’s Rule of five which is an indication of druggability.

**Table 4:** Molinspiration’s estimations of the parameters of Lipinski’s rule of five

Ligands	Log*P*	Molecular weight (g/mol)	noN	nOHNH	Violations	nrotb
5-fluorouracil	0.47	277.24	5	2	0	2
Ifosfamide	2.73	293.77	3	1	0	6

## Discussion

The escalating challenge of drug resistance in the treatment of endometrial cancer necessitates the development of innovative strategies in drug development and therapy optimization. While initial responses to chemotherapy are often favorable, recurrent tumors frequently develop resistance, rendering standard treatments ineffective. This resistance may occur through various mechanisms, including enhanced DNA repair, activation of drug efflux pumps, mutations in drug targets, and dysregulation of oncogenic pathways, such as RAS/MAPK [33, 34]. To address these challenges, a promising strategy involves the rational modification of existing FDA-approved drugs to enhance their therapeutic efficacy while leveraging their established safety profiles.

In this context, 5-Fluorouracil (5-FU), a well-established pyrimidine analog, has been extensively investigated through structure–activity relationship (SAR) research to enhance its anticancer efficacy and reduce off-target toxicity [35]. Specifically, N1 and N3 substitutions have resulted in derivatives with improved selectivity, metabolic stability, and absorption, while minimizing cytotoxic side effects. Furthermore, hybrid molecules of 5-FU combined with amino acids, phospholipids, and polymers have demonstrated potential in improving tumor targeting and pharmacokinetics. Several clinically utilized prodrugs of 5-FU, such as tegafur, capecitabine, and carmofur, further exemplify the utility of this approach in overcoming challenges like poor oral bioavailability and systemic toxicity [36].

In contrast to 5-FU, Ifosfamide, an alkylating agent commonly employed in gynecologic oncology, has received less attention regarding SAR-driven optimization. Nonetheless, research has explored modifications to its chloroethyl side chains to enhance metabolic activation and reduce the formation of toxic by-products, such as acrolein [37]. Prodrug strategies for Ifosfamide are also under investigation to address issues related to nephrotoxicity and resistance. Given the limited but emerging SAR data, our study employed in silico modification and screening to explore novel Ifosfamide analogs that could potentially exhibit improved binding affinity and favorable ADME profiles. Targeted 5-fluorouracil and ifosfamide interactions were studied by docking them with guanosine diphosphate (GDP) bound G12C oncogenic mutant of human GTPase KRAS protein, 4LDJ, and G12C oncogenic variant of human KRAS protein, 4NMM. The drugs, 5-fluorouracil and ifosfamide, were modified in order to improve their efficacy and effectiveness against drug-resistant endometrial cancer. Ifosfamide prevents the cancer cells from dividing and replicating whereas, 5-fluorouracil inhibits the synthesis of deoxythymidine monophosphate (dTMP) which is necessary for the replication and repair of the DNA, thus prevents the endometrial cancer from spreading [28, 29]. While this study is computational, the results provide an initial screening of potential drug candidates. Future experimental validation, including enzyme inhibition assays and cellular studies, will be necessary to confirm these findings.

Binding affinities of both the normal and modified 5-fluorouracil and ifosfamide were compared after docking them with the mutated KRAS protein which is responsible for Type 1 endometrial cancer. Both 5-fluorouracil and ifosfamide had lower binding affinity with mutated proteins of KRAS. Energy absorption of 5-fluorouracil was improved by the addition of methyl group and benzene ring at C-4. Nitrogen atom and methyl group were added at C-3 of additional benzene ring, and fluorine atoms were added at C-4 and C-5 of the same benzene ring, respectively. These types of drug modifications have been previously reported where modification of a drug resulted in an increased activity against the target protein [25, 27]. Ifosfamide was modified by the replacement of nitrogen atom at C-3 with a methyl group and chlorine atom with cyclopentane ring. A similar modification has been reported by [30] in fluoroquinolines to improve their antibacterial and antioxidant activity. Molecular dynamic simulation of the docked complex formed was done through iMODS [31–35]. Simulation analyses confirmed the anti-correlated motion of the most of the amino acids in the conjugate of modified 5-fluorouracil with 4NMM and 4LDJ as well as in the conjugate of modified ifosfamide with 4NMM and 4LDJ. Simulation analysis helped to verify the conjugate formed after the modification of the drugs. Moreover, high Eigenvalues ensured the stability of the conjugates as high amount of energy is required to deform the complexes [38, 39].

SwissADME analysis confirmed the solubility of the modified drugs making them a potential candidate for the treatment of uterine cancer. Solubility of water ensured easy handling and formulation of the drugs. The lipophilicity of modified 5-fluorouracil was optimum, while the lipophilicity of modified ifosfamide was ideal. Both the drugs were unable to penetrate the skin. Ifosfamide can cross the blood–brain barrier (BBB) so it might have adverse effects such as growth of tumor cells and can also affect the central nervous system. However, ifosfamide is used for treating different malignancies in combination with different drugs to reduce its side effects. The side effects can also be reduced by decreasing the dose of administration of ifosfamide. Both the drugs had high GI absorption resulting in a rapid onset of action and higher bioavailability of the drugs of 0.55 fraction (F). Synthetic accessibility of ifosfamide was 5.15, whereas 5-fluorouracil’s synthetic accessibility was 4.58. Molinspiration cheminformatics tool confirmed that there was no violation of Lipinski’s Rule of five by both the drugs. ADMET analysis of 5-fluorouracil makes it a suitable candidate for treating Type 1 endometrial cancer as it is unable to cross the BBB and have better efficacy with the mutated proteins of KRAS gene compared to ifosfamide. ADMET predictions provide an initial assessment of the drug-likeness and pharmacokinetic properties of the modified compounds.

## Limitations and future directions

This study is based solely on in silico analyses and computational modeling, without accompanying in vitro or in vivo experimental validation. The work was conducted as part of an undergraduate research project without access to laboratory facilities or dedicated research funding. While the computational findings provide valuable preliminary insights and hypotheses regarding the therapeutic potential of modified compounds against endometrial cancer, experimental validation through cell-based assays, cytotoxicity testing, and gene/protein expression studies is essential for future research. We hope that these findings will serve as a foundation for further experimental investigations to confirm and extend the computational predictions presented here.

## Conclusion

Successful modification of 5-fluorouracil and ifosfamide improved the energy absorption when they were docked with guanosine diphosphate (GDP)-bound G12C oncogenic mutant of Human GTPase KRAS protein, 4LDJ, and G12C oncogenic variant of Human KRAS protein, 4NMM. It was observed that 5-fluorouracil which is a pyrimidine analog of uracil could serve as a potential candidate for treating Type 1 endometrial cancer. However, pre-clinical and clinical trials are required before using it on humans to remove the biological uncertainty concerning the modification. In case of failure of 5-fluorouracil during pre-clinical testing, ifosfamide can be given as an alternative drug. There is a need to identify functional groups that can improve the efficiency of the drug, decrease administration dosage, and can help to reduce their side effects.

## Data Availability

No datasets were generated or analyzed during the current study.
